# Axon fasciculation in the developing olfactory nerve

**DOI:** 10.1186/1749-8104-5-20

**Published:** 2010-08-19

**Authors:** Alexandra M Miller, Lydia R Maurer, Dong-Jing Zou, Stuart Firestein, Charles A Greer

**Affiliations:** 1 Department of Neurosurgery, Yale University School of Medicine, 333 Cedar Street, New Haven, CT 06520, USA; 2 The Interdepartmental Neuroscience Program, Yale University School of Medicine, 333 Cedar Street, New Haven, CT 06520, USA; 3 Medical Scientist Training Program, Yale University School of Medicine, 333 Cedar Street, New Haven, CT 06520, USA; 4 Department of Biological Sciences, Columbia University, 923 Fairchild Center, New York, NY 10027, USA; 5 Department of Neurobiology, Yale University School of Medicine, 333 Cedar Street, New Haven, CT 06520, USA

## Abstract

Olfactory sensory neuron (OSN) axons exit the olfactory epithelium (OE) and extend toward the olfactory bulb (OB) where they coalesce into glomeruli. Each OSN expresses only 1 of approximately 1,200 odor receptors (ORs). OSNs expressing the same OR are distributed in restricted zones of the OE. However, within a zone, the OSNs expressing a specific OR are not contiguous - distribution appears stochastic. Upon reaching the OB the OSN axons expressing the same OR reproducibly coalesce into two to three glomeruli. While ORs appear necessary for appropriate convergence of axons, a variety of adhesion associated molecules and activity-dependent mechanisms are also implicated. Recent data suggest pre-target OSN axon sorting may influence glomerular convergence. Here, using regional and OR-specific markers, we addressed the spatio-temporal properties associated with the onset of homotypic fasciculation in embryonic mice and assessed the degree to which subpopulations of axons remain segregated as they extend toward the nascent OB. We show that immediately upon crossing the basal lamina, axons uniformly turn sharply, usually at an approximately 90° angle toward the OB. Molecularly defined subpopulations of axons show evidence of spatial segregation within the nascent nerve by embryonic day 12, within 48 hours of the first OSN axons crossing the basal lamina, but at least 72 hours before synapse formation in the developing OB. Homotypic fasciculation of OSN axons expressing the same OR appears to be a hierarchical process. While regional segregation occurs in the mesenchyme, the final convergence of OR-specific subpopulations does not occur until the axons reach the inner nerve layer of the OB.

## Background

In the adult mouse olfactory system, there is a precise topographic organization between the olfactory epithelium (OE) and the olfactory bulb (OB). Regionally defined markers, such as olfactory cell adhesion molecule (OCAM), discriminate between olfactory sensory neuron (OSN) axons innervating the dorsal and ventral domains in the OB, while the final convergence of OSN axons into glomeruli reflects odor receptor (OR) expression [1-4]. However, the spatio-temporal correlates related to the segregation of subpopulations of OSN axons within the developing olfactory nerve remain unknown.

The initial development of the primary olfactory pathway, from the OE to the OB, begins at embryonic day (E)9 with the differentiation of neurons within the olfactory placode (OP). The first OSN axons that cross the basal lamina of the developing OE are seen at E10 to E10.5 [5]; at later ages these coalesce into fascicles surrounded by presumptive olfactory ensheathing cells. The OSN axons appear to follow a scaffold of migrating neurons that emerge from the OP beginning at E10. In what is collectively termed the migratory mass (MM), the OSN axons intermingle with these migrating neurons as they extend towards the presumptive OB. OSN axons first contact and penetrate the telencephalic vesicle on approximately E11 [5-7]. However, most OSN axons remain restricted to the presumptive olfactory nerve layer (pONL) until E15 when glomerulogenesis begins. This phase, the 'waiting period', is similar to that seen for thalamocortical axons in the subcortical plate and may contribute to the segregation of molecularly distinct subpopulations of OSN axons [8].

Each mouse OSN expresses only 1 of approximately 1,200 OR genes, and axons from OSNs expressing the same OR coalesce in two to three glomeruli per OB, typically one lateral and one medial glomerulus [9-12]. Consistent with a possible role in axon-axon interactions, ORs are expressed in OSN growth cones where they may contribute to ligand-induced Ca^2+ ^transients [13,14]. In the absence of an OR, axons fail to converge to the appropriate glomerulus [11,12,15] while substitution of a different OR partially redirects axon coalescence [15,16]. Similarly, changes in functional activity and the expression of adhesion molecules can influence axon coalescence, independent of OR expression [17-19]. Thus, a consensus is emerging that OSN axon coalescence and/or targeting may be a hierarchical process in which multiple mechanisms determine regional positioning in the OB, while OR expression underlies the final convergence of axons within the inner nerve layer and the formation of glomeruli [8].

Recent work introduced the concept of pre-target axon sorting - a gross order and organization within an axon bundle prior to contacting the target - as a potential mechanism in OSN axon coalescence [1,2], but there is continuing controversy about the application of this notion to the olfactory nerve, or the spatio-temporal correlates of axon sorting that would be necessary during early embryonic development.

Here, we show that segregation of subpopulations of axons within the developing olfactory nerve is evident by E12. Using both regional and OR-specific markers, we demonstrate that molecularly homotypic axons follow parallel courses through the mesenchyme, prior to the formation of the cribriform plate, and maintain a spatial congruency as they begin to establish a primitive nerve layer in the developing OB. Homotypic axon fasciculation appears to be a hierarchical process. While regional segregation is prominent within the olfactory nerve, convergence of OR-specific subpopulations of OSN axons, and the initiation of glomerulogenesis, do not occur until the axons cross into the inner nerve layer of the OB.

## Materials and methods

### Animals

Mice were used as follows: pregnant, time-mated CD-1 (Charles River, Wilmington, MA, USA), AC3 knockout heterozygous [19-21]; M72-GFP and P2-LacZ mice (gift from Dr Peter Mombaerts). The M72-GFP mice and the P2-lacZ mice were crossed to create a new heterozygous line containing constructs for both P2 and M72. For the genetically engineered lines, embryonic mice were derived from timed-pregnant females (day of the vaginal plug = E0), which were euthanized using CO_2_, followed by cervical dislocation. Embryonic and postnatal pups were swiftly decapitated and placed in 4% paraformaldehyde in phosphate-buffered saline (PBS; 0.1 M phosphate buffer and 0.9% NaCl, pH 7.4) at 4°C overnight. After fixation, tissue was washed in PBS overnight. Neonatal and embryonic tissue was cryoprotected by immersion in 30% sucrose in PBS at 4°C until the tissue sank, embedded in OCT compound (Tissue-Tek; Miles Laboratories Inc., Elkhart, IN, USA), and frozen in a slurry of dry ice and ethanol. Embryonic specimens were sagittally or coronally sectioned at 20 μm using a cryostat (Reichert-Jung 2800 Frigocut E). Sections were thaw mounted onto SuperFrost Plus microscope slides (Fisher Scientific, Pittsburgh, PA, USA), air-dried, and stored at -20°C until needed. All procedures undertaken in this study were approved by the Animal Care and Use Committees of Yale University and conformed to NIH guidelines.

### Immunohistochemistry and confocal microscopy

The 20 μm cryostat sections were immunostained with antibodies as described and are characterized in Table 1 (n = minimum of 3 for each condition). Briefly, tissue was thawed, air dried, and pre-incubated with 2% bovine serum albumin (Sigma Chemical Co., St Louis, MO, USA) in PBS-T (PBS with 0.3% Triton X-100; Sigma) for 30 minutes to block nonspecific binding sites. Tissue was then incubated with two or three of the primary antibodies detailed in Table 1, diluted as a cocktail in blocking solution, overnight at room temperature. Sections were washed three times in PBS-T for 5 minutes and incubated in secondary antibodies conjugated to Alexa Fluors or cyanide-dyes diluted in blocking buffer for 1 hour at room temperature (Molecular Probes, Eugene, OR, USA, or Jackson ImmunoResearch Laboratories, West Grove, PA, USA; Table 2). Sections were washed (as above), rinsed in PBS, mounted in Gel/Mount mounting medium (Biomeda, Foster City, CA, USA), and coverslipped. To verify the specificity of the antibodies, dilution series were performed and tissue sections were processed in the absence of the primary antibody (data not shown). In all cases, the specificity of the staining was consistent with prior reports in the literature.

**Table 1:** Primary antibodies

Primary antibodies	Company	Species	Dilution
DBA (biotin conjugated)	Sigma	NA	1:100
GAP-43	Novus Biologicals	Rabbit	1:1,000
NCAM	Millipore	Rat	1:1,000
NQO1	Abcam	Goat	1:1,500
NP-1	R&D Systems	Goat	1:750
OCAM	Gift from Y Yoshihara	Rabbit	1:500
DBA (Biotinylated)	Vector Laboratories	NA	1:50
WFA (fluorescein conjugated)	EY Laboratories	NA	1:50
Robo-2	Gift from J Cloutier	Rabbit	1:350
GFP	Abcam	Chicken	1:1,000
β-Galactosidase	Abcam	Rabbit	1:2,000

DBA, *Dolichos biflorus *agglutinin; GAP-43, growth-associated protein 43; GFP, green fluorescent protein; NA, not applicable; NCAM, neural cell adhesion molecule; NQO1, NAD(P)H:quinone oxidoreductase; NP-1, neuropilin-1; OCAM, olfactory cell adhesion molecule; WFA, *Wisteria floribunda *agglutinin.

**Table 2:** Secondary antibodies

Secondary antibodies	Company	Dilution
Dk α Rb 555/488	Molecular Probes	1:1,000
Dk α Gt 488/555	Molecular Probes	1:1,000
Dk α Rt Cy3	Jackson	1:200
Dk α Ch Cy3	Jackson	1:200
Alexa-488-conjugated streptavidin	Molecular Probes	1:200
DRAQ5	Alexis Biochemicals	1:1,000
DAPI	Molecular Probes	1:1,000

Images were acquired with a Leica confocal microscope, using 20×, 40× or 63× objectives, the latter two oil-immersion. Digital images were collected from a single optical plane, approximately 1 μm thick. Digital images were color balanced using Adobe Photoshop CS3 (Adobe Systems, San Jose, CA, USA). The composition of the images was not altered in any way. Plates were constructed using Corel Draw 10.0 for the Macintosh (Corel, Ottawa, Ontario, Canada).

### Three-dimensional reconstructions

Serial 20-μm sections were imaged using an Olympus BX51 Epifluorescent Microscope. The olfactory nerve pathway was reconstructed from the serial sections using IMARIS software (Bitplane AG, St. Paul, MN, USA). Due to the thickness of the sections, the z-parameter needed to be artificially set so that the reconstruction appears continuous. Hence, the x- and y-axes are proportional, while the z-axis is set to maximize viewing of the images. When surfaces were rendered, the colocalization function was used to illustrate the expression patterns of neuropilin 1 (NRP-1) and neural cell adhesion molecule (NCAM).

### Quantitative analysis

Quantitative analyses were performed on timed-pregnant M72/P2 embryos at E16. Images were rotated so that axon fascicles were parallel to the longitudinal axis of the image. The distance across the entire fascicle was defined on the basis of NCAM staining. The width of each M72 or P2 fascicle was defined based on lacZ or GFP staining. The distance of the M72/P2 axons was defined as the outer point on the leftmost fascicle to the outer point of the rightmost fascicle. The distance was measured using the Measure and Label function in Image J.

## Results

### Development of the olfactory nerve

The OE arises from the OP, a specialized epithelial thickening in the rostro-lateral aspect of the head. The OP is separated from the telencephalon by the frontonasal mesenchyme. The interactions between the OP and the mesenchyme are necessary for the proper development of the molecular and cellular diversity in the OE as well as the establishment of the axon trajectories that comprise the olfactory nerve [22]. The adult olfactory nerve includes over approximately 1,200 subpopulations of axons, each representative of 1 from a candidate genome of 1,200 different ORs. Additionally, there are larger subpopulations of axons that express regional markers, including, but not limited to, NAD(P)H:quinone oxidoreductase 1 (NQO1), OCAM, NRP-1, Roundabout2 (Robo2), and various cell surface carbohydrates. While NRP-1, Robo2, and some cell surface carbohydrates have been implicated in axon targeting and the proper formation of the olfactory nerve, other molecules such as NQO1 and OCAM have not yet been shown to play a functional role related to axon targeting in the OB. Nonetheless, these markers are effective as tools for testing hypotheses regarding the molecularly homotypic convergence of subpopulations of OSN axons during the formation of the olfactory nerve.

Before assessing the behavior of subpopulations of axons as the olfactory nerve forms, it is critical to understand the initial developmental events that give rise to the primary afferent pathway. Cells first differentiate and express neuronal markers, such as β-tubulin III, within the OP at E9 (AM Miller *et al*., submitted). Subsequently, by E10, two different populations of neurons differentiate within the OP, the OSNs that will remain within the OE, and those neurons that will exit the OE and join the MM. The MM cells migrate out by E10, prior to the outgrowth of OSN axons, and coalesce to form a scaffold that extends toward the as yet undifferentiated basal telencephalon (AM Miller *et al*., submitted). Beginning at E10 to E10.5 in mice, the OSN axons extend across the basal lamina [5] and join the scaffold of MM cells, turning towards the telencephalon [23,24]. Initially, the trajectories of MM cells and the OSN axons rely on a combination of guidance cues within the mesenchyme, axon-axon interactions, and telencephalic-derived chemotropic factors [25,26]. By E11.5 the OSN axons contact the rostral-most tip of the telencephalon and innervate the presumptive OB [25] (AM Miller *et al*., submitted). The first synapses are detected in the OB at E14, but are not found in high numbers until E15 at the onset of glomerulogenesis [8,27,28].

**Figure 1:**
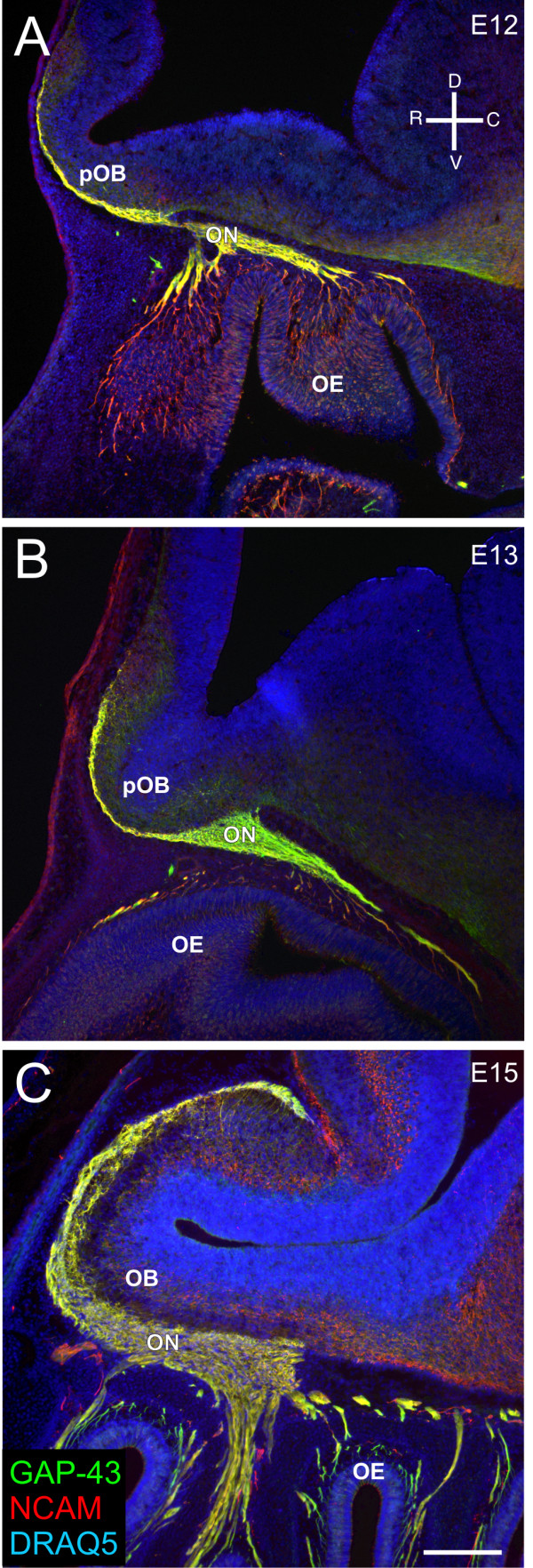
**Development of the olfactory nerve pathway**. **(A-C) **Sagittal sections of CD1 embryos stained for GAP-43 (green)/NCAM (red)/DRAQ5 (blue) at E12 (A), E13 (B), and E15 (C). An orientation compass is shown in (A). Scale bars = 200 μm. OE, olfactory epithelium; ON, olfactory nerve; pOB, presumptive olfactory bulb.

Using neuronal markers, GAP43 (growth-associated protein 43), a marker of immature neurons, and NCAM, an adhesion molecule, we imaged the development of the olfactory nerve in sagittal sections at E12, E13, and E15. At E12, the axons extend out of the OE and into the mesenchyme (Figure 1A). As they transverse the mesenchyme, they begin to fasciculate, forming the presumptive olfactory nerve. The axons wrap the nascent OB, forming a pONL by E12, but most do not yet penetrate the basal lamina of the presumptive OB (Figure 1A). At E13, the nerve thickens, but the axons remain in the pONL outside the basal lamina of the telencephalon (Figure 1B). At E13 the increasing flexure of the telencephalic vesicle foreshadows the nascent OB (Figure 1B). By E15, the shape of the telencephalon has changed dramatically, the walls have thickened and the flexure is more pronounced (Figure 1C). Additionally, the nerve has grown significantly and axons have extended into the OB, penetrating the basal lamina. It was previously suggested that prior to penetrating the OB, during the 'waiting period', between E12 and E15, OSN axons were organizing into molecularly defined subpopulations prior to coalescence into glomeruli [8]. We propose an alternative hypothesis - that sorting begins prior to E12 and proceeds within the mesenchyme.

### Homotypic regional axon segregation begins in the mesenchyme

**Figure 2:**
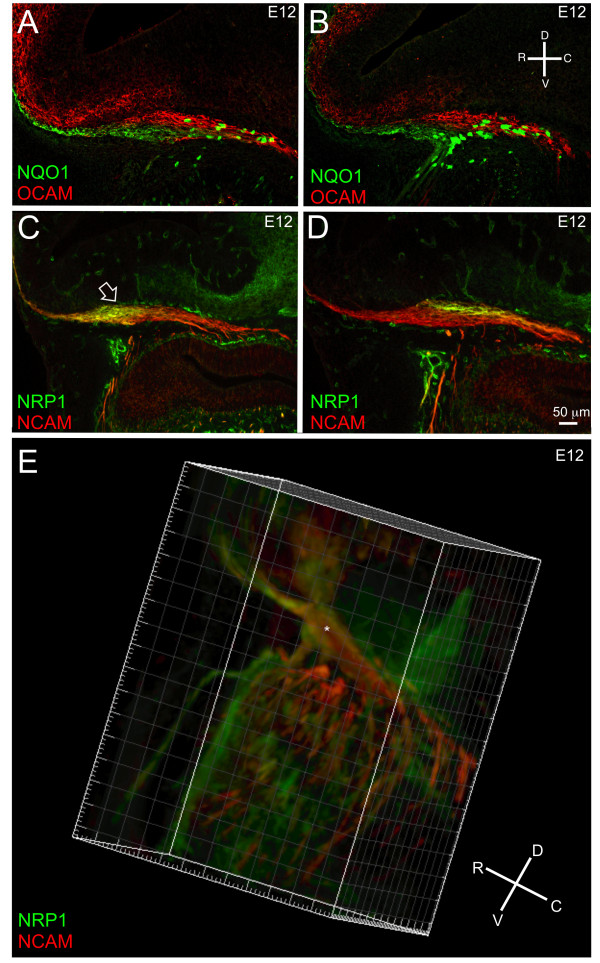
**Homotypic axon segregation occurs by E12**. Sagittal sections of CD1 embryos. **(A, B**) NQO1 (green)/OCAM (red). NQO1^+ ^cells are clustered caudally, mostly within the OCAM^+ ^region of the nerve. **(C**-**E) **NRP-1 (green)/NCAM (red): (C) lateral section - open arrowhead indicates NRP-1^+ ^region; (D) medial section; (E) three-dimensional reconstruction from the lateral side of the nerve. The asterisk indicates the central region of the nerve devoid of NRP-1 staining. An orientation compass is shown in (B), for (A-D) and in (E). Scale bar shown in (D) = 50 μm in (A, B) and 100 μm in (C, D).

In the adult OB, regional organization of OSN axons is well-documented. However, the temporal and spatial correlates of axon segregation within the developing nerve are not established. We asked the following two questions: at what age is regional/molecular specificity established within the developing nerve; and where along the olfactory nerve pathway do homotypic axons begin to fasciculate? To track regional segregation, we used NQO1 (OE zone 1 OSNs projecting to the dorsomedial OB) [29] and OCAM (OE zones 2 to 4 OSNs projecting to the ventrolateral OB) [3,30]. At E12, just 48 hours after the first extension of axons across the basal lamina, axons expressing NQO1 (green) and OCAM (red) segregate in topographically distinct sections of the nerve (Figure 2A, B).

NQO1^+ ^axons are topographically restricted to the rostral and ventral aspects of the nerve, whereas the OCAM^+ ^axons track caudally and dorsally (Figure 2A, B). The expression pattern of NQO1 and OCAM did not vary significantly along the medial-lateral axis. Interestingly, a population of cells expressing NQO1 also appears in the nerve, primarily in the caudal aspect. These cells are a subset of MM cells (AM Miller *et al*., submitted). While the cells comprising the MM emerge from the OP as early as E10, there is no evidence of the NQO1^+ ^population until E12.

Next, we assessed the topography of axons expressing NRP-1, the ligand-binding subunit for Semaphorin 3A [31-34]. NRP-1 is expressed by a subset of OSNs whose axons terminate in medial and lateral glomeruli [35]. Unlike NQO1 and OCAM, which are expressed in distinct zones within the OE, NRP-1 does not conform to zonal patterning in the OE [2].

Axons expressing NRP-1 occupy a spatially distinct region in the nerve. Laterally, NRP-1 is most strongly expressed rostrally (Figure 2C, open arrow; see Additional file 1 for single channel localization), but medially, expression shifts caudally (Figure 2D; Additional file 1). A rostral-to-caudal transition zone can be recognized at the lateral-medial midline. To better visualize this segregation, we created a three-dimensional reconstruction of the olfactory nerve. In a lateral view, NRP-1 expression is visible in the rostral nerve and axon bundles (Figure 2E; Additional file 2). The axon fascicles targeting the central and caudal aspects of the nerve are primarily NRP-1^-^/NCAM^+^. Similarly, there is a region that is consistently NRP-1^- ^(NCAM^+^; red) in the central-most aspect of the nerve (Figure 2E, asterisk; Additional file 2). NRP-1 also stains blood vessels, which follow a trajectory parallel to the axons extending up from the OE.

Next, we rendered surfaces onto the same three-dimensional reconstruction using the colocalization feature on IMARIS to assess the segregation between the NRP-1^+^/NCAM^+ ^regions (yellow) and the NRP-1^-^/NCAM^+ ^regions (red; Additional file 2). For clarity, the NRP-1^+^/NCAM^- ^area, which stains the mesenchyme and blood vessels only, was not included in the reconstruction. In a medial view (Additional file 3A), axons expressing NRP-1 are seen traversing the nerve rostral-to-caudal to join the caudal aspect of the nerve where NRP-1^+ ^axons are located. Laterally, as discussed above, there is a central/ventral portion of the nerve that remains NRP-1^-^. Medially, strong NRP-1 expression is seen rostrally. Laterally (Additional file 3B), NRP-1 expression is segregated to the rostral region of the nerve, and the associated fascicles. Generally, in the caudal region, there is an absence of NRP-1 expression. These segregation patterns are consistent with the confocal images displayed above, and reinforce the notion that NRP-1^+ ^axons sort in a consistent, spatially defined manner.

**Figure 3:**
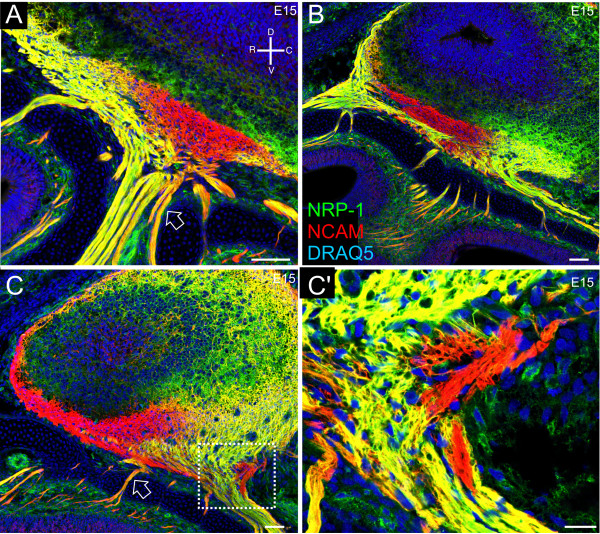
**At E15, axons expressing NRP-1 continue to distinctly compartmentalize in the olfactory nerve.** Sagittal sections of CD1 embryos. **(A-C') **NRP-1 (green)/NCAM (red)/DRAQ5 (blue). (A) Lateral section. The open arrow indicates lack of segregation in fascicles targeting the caudal nerve. (B) Lateral-medial section. (C) Medial section. The open arrowhead shows the lack of segregation in rostral fascicles coming up to join the nerve. (C') High magnification of boxed region in (C). An orientation compass is shown in (A). Scale bars in (A-C) = 50 μm, C' = 10 μm.

This distinct segregation, as shown with NRP-1, persists at later embryonic ages, as the nerve becomes increasingly well defined. When fasciculation begins, just after individual axons cross the basal lamina, segregation of homotypic subpopulations has not yet occurred. This is seen in Figure 3A-C where the integration and close apposition of NRP-1^+^/NCAM^+ ^axons and NRP-1^-^/NCAM^+ ^axons (orange) is found in the lamina propria, proximal to the OE (Figure 3A; open arrow). The axons coming from the rostral epithelium appear to segregate prior to those coming from the caudal region (Figure 3A). Laterally, NRP-1 expression is primarily rostral (Figure 3A). In sagittal sections, there is only a small patch of NRP-1 expression in the caudal tip of the nerve. In more medial areas of the nerve, NRP-1 expression is both rostral and caudal (Figure 3B). NRP-1^+ ^axons in the most ventral aspect of the nerve are most likely those crossing from rostral-to-caudal as they travel medially [35]. Other than this small group of NRP-1^+ ^axons, as is the case at E12, the central region of the olfactory nerve is devoid of NRP-1 expression. The axons extending up to the caudal region of the nerve are NRP-1^+^, and distinct segregation is apparent as the fascicles join the nerve. NRP-1 also stains the deeper layers of the OB, blood vessels, and mesenchyme (Figure 3A-C). Medially, NRP-1 expression is most prominent in the caudal region of the nerve (Figure 3C), but there are a few mixed fascicles (orange) in the rostral area that are NRP-1^+ ^(Figure 3C; open arrow). The dorsomedial aspect of the nerve is NRP-1^+-^, and as the axons fasciculate and travel from the lamina propria toward the nerve, there is a caudal section that remains NRP-1^- ^(Figure 3C, C').

**Figure 4:**
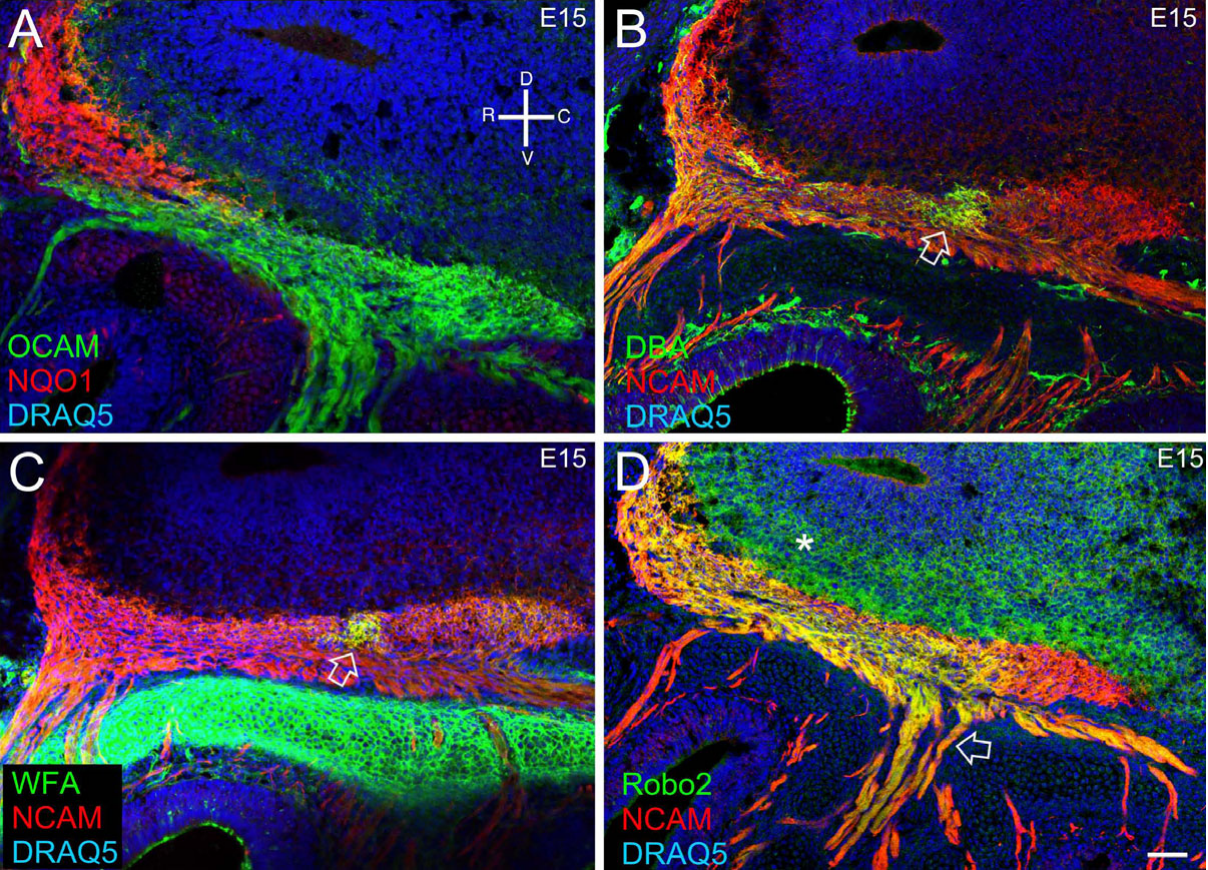
**At E15, established regional markers show that pre-target axon sorting in the olfactory nerve occurs early in development.** Sagittal sections of CD1 embryos. All panels are stained with the nuclear marker DRAQ5 (blue). **(A) **NQO1 (red)/OCAM (green). **(B) ***Dolichos biflorus *agglutinin (DBA; green)/NCAM (red). Open arrow indicates focal DBA positive axons. **(C) ***Wisteria floribunda *agglutinin (WFA; green)/NCAM (red). Open arrow indicates focal WFA positive axons. **(D) **Robo2 (green)/NCAM (red). Open arrow indicates Robo2/NCAM positive fascicle. Asterisk indicates Robo2 staining in the mitral cell and external plexiform layers of the olfactory bulb. An orientation compass is shown in (A). Scale bars shown in D = 100 μm for (A-D).

At E15, NQO1 and OCAM occupy the same regions of the nerve as shown at E12 (Figure 4A), with NQO1 in the dorsal-rostral area, and OCAM ventral-caudal. The boundary between the two regions is definitive, with no colocalization or overlap. At this age, the NQO1^+ ^MM cells are no longer seen. To further this analysis, we turned to other regional markers to assess whether definitive boundaries are a ubiquitous feature of the developing nerve.

Two plant lectins implicated in axon guidance, *Dolichos biflorus *agglutinin (DBA) and *Wisteria floribunda *agglutinin (WFA), bind to subpopulations of cell surface carbohydrates expressed by subsets of OSNs. In the adult mouse OB, DBA primarily labels dorsal glomeruli [36,37]. WFA is expressed by E12.5, and at E15.5 is present in a subset of axons that terminate in the dorsal main OB [38].

In the nascent nerve, DBA labels a subset of axons in a dorsal patch midway across the olfactory nerve pathway (Figure 4B; open arrow). There is also diffuse DBA staining in the rostral region of the nerve, as well as a small patch that is strongly DBA^+ ^in the rostral/dorsal part of the nerve (Figure 4B). The caudal aspect of the nerve has the lowest level of staining (Figure 4B). WFA expression was also strongest in a patch in the dorsal part of the nerve, midway across (Figure 4C; open arrow). Its pattern of expression appears similar to that of DBA. This was expected because early postnatal expression of DBA and WFA is overlapping (AMM and CAG, unpublished data). In the fascicles entering the nerve rostrally, colocalization or overlap of WFA^+^/NCAM^+ ^axons is found. In the nerve itself, however, staining is strongest in the described dorsal patch, with lower levels caudally (Figure 4C).

Robo2, a well-established axon guidance molecule, has a graded pattern of expression in the OE, high dorsomedial and low ventrolateral [39]. This regional pattern is also found in the OB, where Robo2 is most strongly expressed in the OSN axons that project to the dorsomedial aspect. Similarly, Robo2 is expressed in a gradient-like pattern in the olfactory nerve (Figure 4D). The gradient-like pattern is apparent going from the rostral-to-caudal aspect of the nerve. The caudal end is devoid of Robo2 staining (Figure 4D). Axon fascicles coming from the basal lamina to target the middle-to-caudal end of the nerve do not show distinct segregation, appearing orange (Figure 4D, open arrow). Robo2 staining also appears in the mitral cell and external plexiform layers of the OB (Figure 4D, asterisk). While these regional markers demonstrate unique segregation patterns, each also illustrates that axons coursing towards different regions in the OB are segregating within the mesenchyme. Because some of the markers we have used to assess axon sorting, such as NRP-1, do not shown evidence of zonal segregation within the OE, these data suggest that developmental pre-target OSN axon sorting within the mesenchyme is important in establishing the topography between the OE and OB.

### Segregation of regional markers in the olfactory nerve pathway is altered in the absence of adenylyl cyclase 3

**Figure 5:**
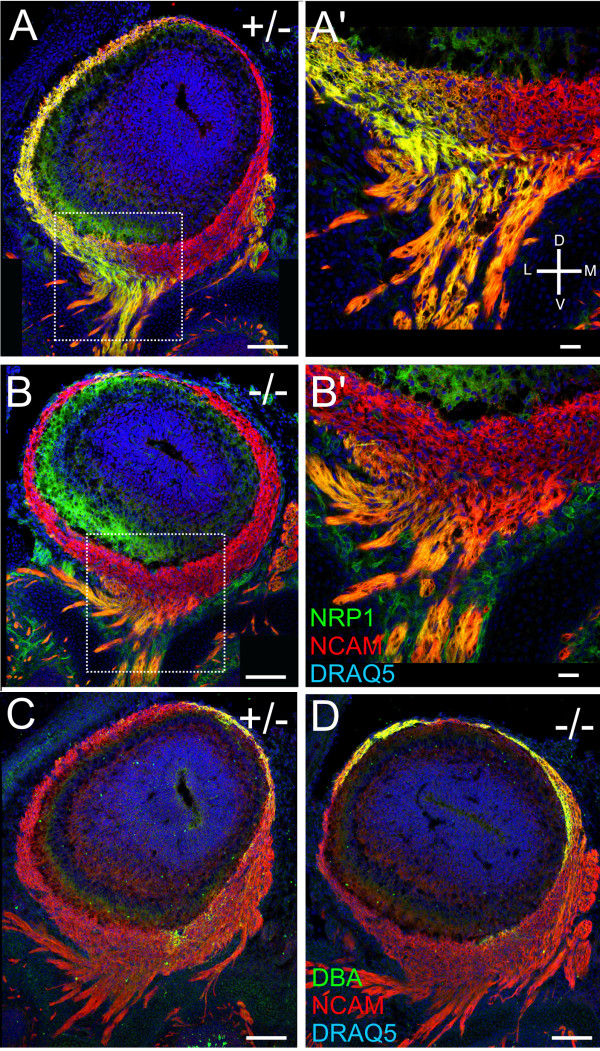
**Levels of NRP-1 and DBA expression are changed in the absence of adenylyl cyclase 3**. **(A-D) **Coronal sections from AC3 heterozygous versus homozygous knock-out mice at E15. (A, B) NRP-1 (green)/NCAM (red)/DRAQ5 (blue); (C, D) DBA(green). (A, A') Coronal cross-section of the OB from a heterozygous AC3 mouse stained for NRP-1. (B-B') Coronal section from a homozygous AC3 knockout mouse stained for NRP-1. (A') High-magnification of boxed area in (A). (B') High-magnification of boxed area in (B). (C) Coronal section of the OB from a heterozygous AC3 knockout mouse stained for DBA. (D) Coronal section from a homozygous AC3 knockout mouse stained for DBA. An orientation compass is shown in (A'). Scale bars = 100 μm in (A-D) and 25 μm in (A'-D').

Previous work established that perturbation of the cAMP pathway alters axon convergence and the fine structural organization of glomeruli [2,17,19,20]. To determine if regional organization within the embryonic nerve, or the sorting of axons within the mesenchyme, is affected by functional activity, we used the adenylyl cyclase 3 (AC3) knockout (KO) mouse. We compared the expression patterns of NRP-1^+ ^and DBA^+ ^axons in AC3 heterozygous versus homozygous KO mice. In E15 heterozygous AC3 mice, rostral NRP-1 staining is strong laterally, and absent medially (Figure 5A; see Additional file 4 for single channel localization), as we found in the CD1 mice. As the fascicles enter the nerve layer, they distinctly segregate (Figure 5A, A'; Additional file 4). However, small axon fascicles within the lamina propria and the larger fascicles connecting to the nerve layer of the OB appear to contain both NRP-1^+ ^and NRP-1^- ^axons, making the fascicles appear orange. These observations were consistent with the patterns seen in the CD-1 mice.

The expression patterns of NRP-1 in the AC3 KO mouse are significantly different. In the nerve layer of the OB, NRP-1 expression is very weak in the ventral axon fascicles contacting the OB (Figure 5B, B'; Additional file 4). However, the deeper layers of the OB, including the external plexiform layer, have strong lateral NRP-1 expression that is more prominent than in the heterozygotes (Figure 5A versus 5B; Additional file 4). Instead of exhibiting distinct segregation as the axons enter the nerve layer, NRP-1 expression diminishes, and only NCAM staining can be observed (Figure 5B'; Additional file 4). In the axon bundles, while low NRP-1 expression is evident by light orange bundles, it is notably less than that of the heterozygote (Figure 5B, B'; Additional file 4).

The expression patterns of DBA in the AC3 heterozygotes versus the homozygous KO mice provides a clearer picture of the altered spatial segregation of axons. As described earlier, DBA expression is typically restricted in a subset of axons in the most dorsal aspect of the OB [36,37] (Figure 5C; Additional file 4). However, in the homozygous AC3 KO mice, DBA staining covered a broader area extending fully across the dorsal aspect of the developing OB, from midline medial to midline lateral (Figure 5D; Additional file 4). In summary, by examining both DBA and NRP-1 levels and expression patterns, we can conclude that the aberrant glomerular targeting in the AC3 mice is likely a manifestation of improper regional segregation within the developing olfactory nerve pathway that can be seen as early as E15, at the onset of glomerulogenesis.

### Axon trajectory in OR-specific subpopulations of axons

Next, we turned our attention to OR-specific subpopulations of axons (M72 and P2) to determine how the smallest subsets of homotypic OSN axons behave during the development of the olfactory nerve. To study these OR-specific axon subpopulations, we used genetically engineered homozygous mice, P2-IRES-taulacZ and M72-IRES-tauGFP (kind gift from Dr P Mombaerts) and heterozygous P2-lacZ/M72-GFP mice that we bred to visualize the behavior of the M72^+ ^and the P2^+ ^axon subpopulations at E16, while the olfactory nerve is forming. While M72^+ ^and P2^+ ^OSNs can been seen earlier in these mice, we chose to look at E16 because this was the first age at which there were sufficient numbers of OSNs expressing each marker to assess the interaction and behavior between homotypic (M72:M72; P2:P2) and heterotypic (M72:P2) axons and fascicles.

**Figure 6:**
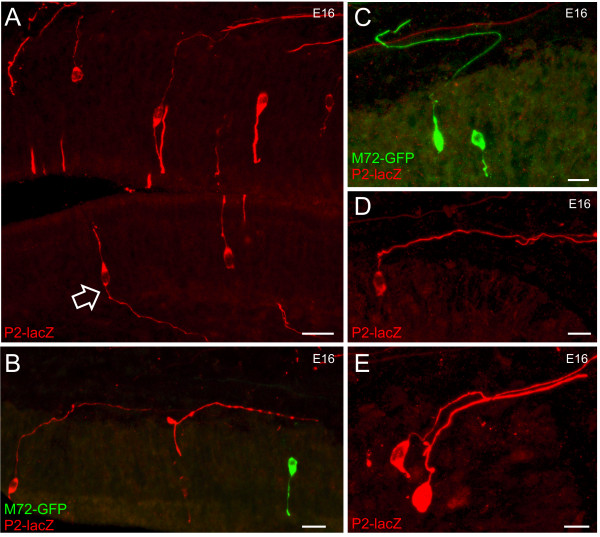
**Trajectory of the OSN axons crossing the basal lamina.** Sagittal sections of P2-lacZ/M72-GFP embryos. **(A-E) **Z-series projections taken from sagittal sections of E16 heterozygous P2-lacZ (red) and M72-GFP (green) mice. (A, D, E) P2 OSNs within the OE. The axons turn sharply towards the bulb as they exit the basal lamina (open arrow in (A)). (B) Three OSNs (two P2, one M72) within the OE. (C) Two M72 axons within the OE. Sometimes the axons initially turn the wrong way and have to correct their trajectory as they travel towards the bulb. Scale bar in (A-E) = 50 μm.

As OSNs differentiate in the OE, they extend a single axon across the basal lamina into the mesenchyme. Almost immediately, most OSN axons make a stereotypic approximately 90° turn in the direction of the olfactory nerve pathway (Figure 6A, B, D, E, open arrow). In some instances axons appear to make mistakes that require several turns to correct (Figure 6C). Interestingly, the axons do not make gradual turns; rather, the turns are bold and definitive, suggesting the presence of boundary cues in the mesenchyme.

**Figure 7:**
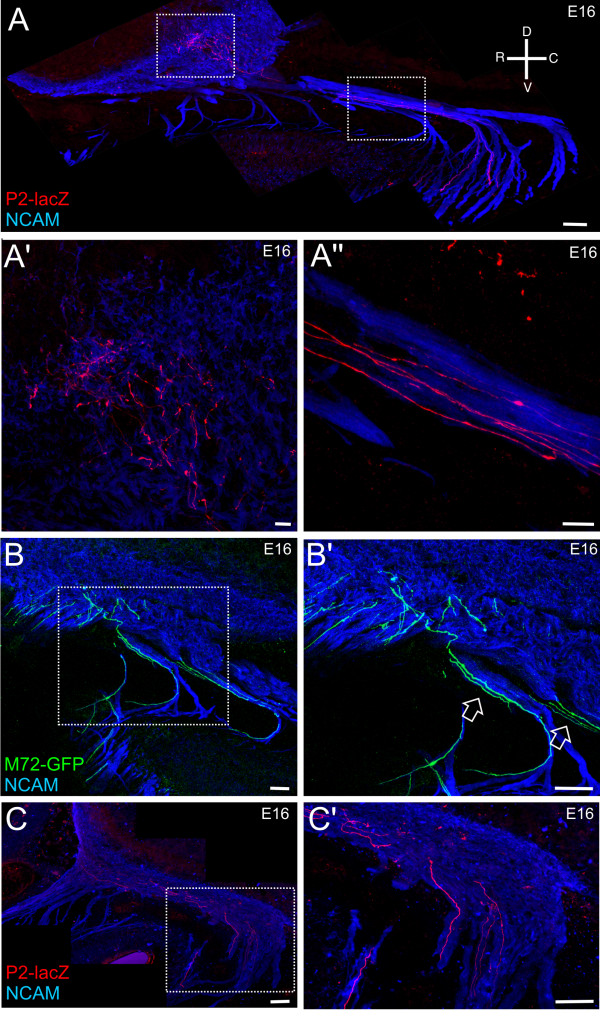
**OR-specific subpopulations of OSN axons show evidence of regional segregation within the olfactory nerve.** Sagittal sections of P2-lacZ/M72-GFP embryos. **(A-C, A'-C') **Z-series projections taken from sagittal sections of E16 P2-lacZ or M72-GFP mice. (A) Low-powered montage of the olfactory nerve pathway of a heterozygous P2-lacZ mouse; (A') high-magnification image of the left-hand boxed area in (A); (A'') high magnification image of the right-hand boxed area in (A), showing the P2^+ ^axons approaching the inner nerve layer of the OB. (B) Low-powered montage of the olfactory nerve pathway of a homozygous M72-GFP mouse; (B') high-magnification image of the boxed area in (B). Open arrowheads indicate incomplete homotypic fasciculation. (C) Low-powered montage of the olfactory nerve pathway of a heterozygous P2-lacZ mouse; (C') high-magnification image of the boxed area in (C). Orientation compass shown in (A). Scale bars in (A, B, B', C, C') = 100 μm and in (A', A'') = 5 μm.

**Figure 8:**
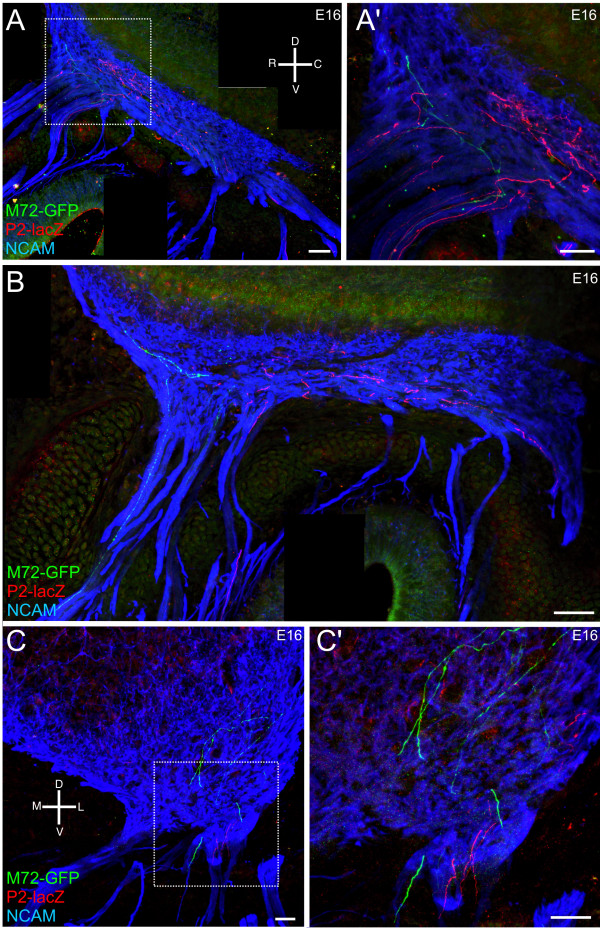
**OR-specific subpopulations are regionally restricted within the olfactory nerve pathway.** Sagittal sections of P2-lacZ/M72-GFP embryos. (A-C) Z-series projections taken from sagittal sections of E16 heterozygous P2-lacZ (red) and M72-GFP (green) mice. (A) Sagittal low-powered montage allowing visualization of two subpopulations of OSNs, P2 (red) and M72 (green) axons in the olfactory nerve; (A') high-magnification image of the boxed area in (A). (B) Sagittal low-powered montage of P2/M72 axons within the nerve. (C) Coronal low-powered image demonstrating the clustering of P2 axons and M72 axons; (C') high-magnification image of the boxed area in (C). An orientation compass is shown in (A) for (A, B) and in (C) for (C, C'). Scale bars = 25 μm.

**Figure 9:**
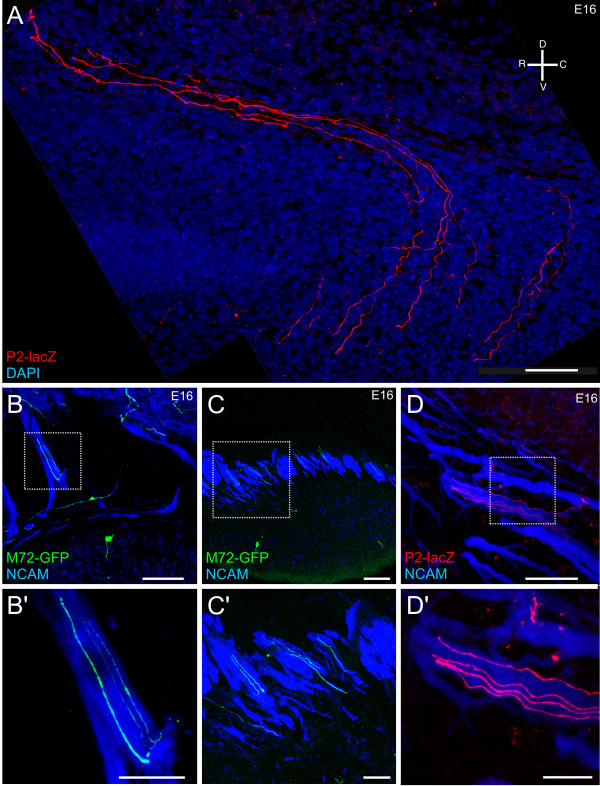
**OSN axon fasciculation in the olfactory nerve pathway.** Sagittal sections of P2-lacZ/M72-GFP embryos. **(A-D) **Z-series projections taken from sagittal sections of E16 P2-lacZ or M72-GFP mice. Low-powered montages of the olfactory nerve pathway of (A) a heterozygous P2-lacZ mouse and (B, C) taken from a homozygous M72-GFP mouse. (B', C') High-magnification view of boxed areas in (B, C). (D) Low-powered montages of the olfactory nerve pathway taken from a P2-lacZ heterozygous mouse; (D') high-magnification view of the boxed area in (D). An orientation compass is shown in (A). Scale bars in (A, B, C, D) = 50 μm and in (B', C', D') = 10 μm.

As OR-specific subpopulations of axons course through the mesenchyme, they travel along a route that appears to be conserved across animals. A subset of P2^+ ^axons enter the olfactory nerve caudally where they join the ventral-most fascicles, and travel along the ventral aspect of the olfactory nerve until they are proximal to the OB, at which point they defasciculate and enter the inner nerve layer (Figure 7A, A'', C, C'; Additional file 5). Another subset of P2^+ ^axons appear to enter rostrally (Figure 8A, A', B). Interestingly, the M72^+ ^axons also appear to travel in rostral-directed OSN fascicles and wrap the most rostral aspect of the OB as they travel dorsally (Figures 7B and 8A, A', B; Additional file 6). Thus, the OR-specific axon subpopulations appear regionally restricted, traveling within the same, limited number of individual fascicles (Figure 7; Additional files 5 and 6). Importantly, while the OR-specific subpopulations are in close regional proximity, often even confined to the same fascicle, individual axons or small fascicles do not completely coalesce despite their intimate proximity (Figures 7 and Figure 9; Additional files 5, 6, 7, 8, 9, and 10). For example, in Figure 7B, B' (open arrows) two different 'subsets' of M72^+ ^axons are seen coursing along parallel trajectories within the olfactory nerve without homotypic fasciculation. While the OR-specific OSN axons are regionally segregated as they transverse the mesenchyme, and when they first exit the basal lamina, they are spread throughout the width of the axon fascicle in which they are traveling. For example, in Figure 9B-D, B'-D' the OR-specific axons occupy up to approximately 90% of the total distance across the fascicle, suggesting that there is not a preferential interaction between axons that express a single OR at this point. However, there is some evidence of incomplete homotypic fasciculation. In Figure 9B, B', the left-most M72^+ ^axon is 0.58 microns in diameter while the two M72^+ ^axons on the right are 0.31 microns, suggesting that at least two M72^+ ^axons have fasciculated to form a mesaxon. Examples of mesaxons that are OR-specific are prevalent throughout the mesenchyme (Figures 7, 8, and 9).

Next, we analyzed heterozygous P2-lacZ/M72-GFP mice to assess the spatial segregation of specific OR-subpopulations expressed in different zones of the OE (P2 is in zone 2 and M72 in zone 1) and projecting to different regions of the OB (P2 axons to the caudal-ventral region of the medial and lateral OB; M72 axons to the caudal-dorsal region of the medial and lateral OB). Generally, the two OR-specific subpopulations remained segregated (Figure 8B, C), though there were instances when single P2^+ ^and M72^+ ^axons appeared closely apposed as they crossed paths (Figure 8A, A'). Isolated axons were also found occasionally in a regional domain defined primarily by the other OR subpopulation (Figure 8C, C').

## Discussion

Here, we: (1) demonstrate that pre-target axon sorting begins as early as embryonic day 12, less than 48 hours after the first OSNs axons have crossed the basal lamina; (2) show that regional sorting occurs as the axons travel through the lamina propria into the mesenchyme, and is clearly evident within the olfactory nerve pathway; (3) illustrate that while regional segregation occurs in the mesenchyme, ultimate coalescence of OR-specific subpopulations does not occur until the OSN axons cross into the inner nerve layer of the bulb; (4) suggest that in the absence of functional activity, pre-target axon sorting is perturbed, which could be the underlying mechanism for the glomerular mistargeting seen in the AC3 KO mice. Based on these data we propose that beginning at the earliest stages of olfactory development, a hierarchical model for homotypic fasciculation can be observed. Regional segregation occurs in the mesenchyme, but OR homotypic axons do not completely fasciculate until they reach the inner nerve layer of the OB.

### Regional homotypic axon segregation occurs in the mesenchyme

At E10, two distinct populations of neurons differentiate within the olfactory placode; the OSNs and the MM cells. Following the migration of the MM cells across the basal lamina, the OSN axons begin to extend across the basal lamina where they come in contact with the MM cells and the ensheathing cells to form clusters that ultimately coalesce as the MM. When the OSN axons move through the lamina propria, into the mesenchyme, they sort according to their molecular phenotype. Clear regional segregation can be visualized with an assortment of markers, including NQO1/OCAM, NRP-1, Robo2, and plant lectins. While our understanding of the functional role of these molecules in the olfactory system remains unclear, several, including NRP-1, Robo2, and the lectins, have been implicated in axon targeting. Given our data, it seems plausible that these and other molecules influence the sorting of OSN axons as they cross the basal lamina. This suggests that the regional topographic organization of the OB is established in the mesenchyme.

### Pre-target axon sorting is perturbed in the absence of AC3

Previous work suggested that altering cAMP expression severely affects axon convergence and glomerular formation [17,19,20]. Axons from OSNs expressing OR I7 exhibit targeting errors and aberrant glomerular formation when cAMP production is decreased. Of further relevance to the data reported here, the OR I7 axons typically label for NRP-1, but in AC3 KO mice, NRP-1 expression is greatly reduced in parallel with the perturbation of I7 axon targeting and glomerular formation [40]. This suggests that the level of NRP-1 expression may be modulated by cAMP and is a determinant of OSN axon coalescence/fasciculation. To explore this hypothesis further, we examined axon fasciculation and the expression of regional markers during embryogenesis in the absence of AC3. Our data showed a clear loss of the distinct regional segregation that normally occurs within the mesenchyme, prior to the axons innervating the OB. While levels of NRP-1 were reduced, minimal expression was seen in the OB nerve layer and axon bundles, unlike the postnatal day 20 mouse [41]. Our data therefore suggest strongly that the aberrant glomerular formation seen in older mice following down-regulation of cAMP is a function of perturbed regional sorting of axons within the embryonic mesenchyme, prior to axons reaching the nascent OB. Moreover, our data are consistent with the plausible hypothesis that one of the molecular mechanisms downstream of cAMP may be NRP-1 expression [2,41].

### Trajectory and organization of OR-specific subpopulations of axons in the developing nerve

We previously demonstrated with high resolution confocal and electron microscopy that the stable homotypic fasciculation of OSN axons expressing the same OR did not necessarily occur prior to the axons entering the inner nerve layer, proximal to the target glomerulus [42]. In many instances axons 'followed tortuous or isolated trajectories before entering the appropriate glomerulus.' However, there was a stark discrepancy in the behavior of OR-specific OSN axons in the outer versus the inner olfactory nerve layer. In the former, most OR-specific axons coursed individually or in small fascicles. As axons entered the inner olfactory nerve layer, they formed larger homotypic fascicles immediately prior to coalescing into a glomerulus [42].

To extend these studies, we investigated the embryonic fasciculation of OR-specific subpopulations of OSN axons. Specifically, we asked if there was evidence of an OR-specific glomerular map within the embryonic mesenchyme. Genetically engineered P2-IRES-taulacZ and M72-IRES-tauGFP and heterozygous P2-lacZ/M72-GFP mice were processed for high-resolution confocal microscopy to examine the organization and fasciculation of OR-specific OSN axons while traversing the mesenchyme.

We found that as OSN axons exit the basal lamina they make a stereotypic approximately 90° turn towards the OB. As the OR-specific subpopulations course through the mesenchyme they are heterotypically fasciculated; M72-GFP axons do not necessarily appose each other and P2-tauLacZ axons do not necessarily appose each other. While in many instances two axons of the same OR subpopulation are in very close proximity, or in some cases apposed, the positioning is not stable. Overall, the rule is that multiple isolated axons, or small fascicles, traverse the mesenchyme following parallel paths within the same larger fascicle, but there is no precedent for them to be adjacent or apposed. This argues against the 'contextual model of self-sorting' proposed by Mombaerts [43] in which the OR, or an OR-containing complex, is responsible for homophilic and heterophilic interactions that promote the sorting of OSN axons into glomeruli. Were this the case, the adhesive forces/interactions between the ORs expressed in the growth cones and along the shaft of the axons would seem likely to promote homotypic fasciculation whenever the axons were in close proximity or in apposition. However, through the mesenchyme our data show that such interactions are transient, without evidence of stable homotypic fasciculation. It may be that the role of the OR is topographically defined and that the nature of the binding partners changes at the time of glomerular coalescence, much as the properties of guidance molecules can change from repulsive to attractive during development (for review see [44,45]).

We have shown that within 48 hours of the first OSN axons crossing the basal lamina of the OE, there is regional, pre-target axon sorting occurring within the mesenchyme. Using a panel of well-established regional markers, we demonstrated that regional segregation occurs in a topographically restricted pattern and is activity-dependent. Interestingly, while the OR-specific subpopulations do show a clear pattern of regional segregation within the nerve, there is no evidence of homotypic OR fasciculation until the OSN axons reach the inner nerve layer of the OB.

## Conclusions

**Figure 10:**
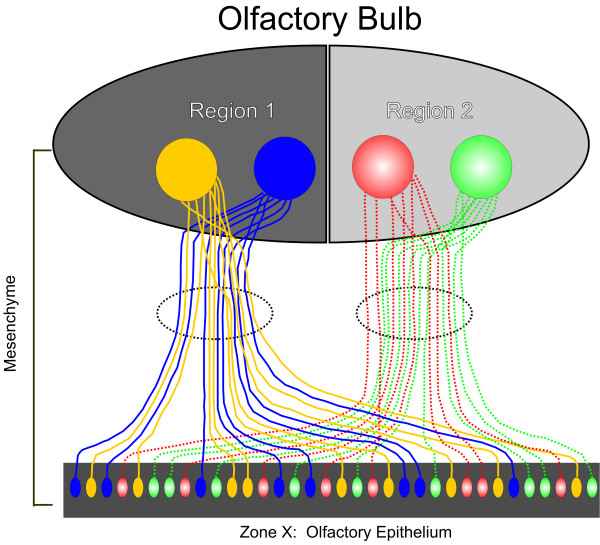
**Hierarchical model of OSN axon sorting**. OR-specific OSNs (each color represents an individual OR) are randomly distributed throughout one of approximately four zones along the dorsal-ventral axis in the OE, with no known organization along the anterior-posterior axis. OSNs each express some currently unknown constellation of guidance molecules (each pattern represents a set of guidance molecules). The OSN axons sort regionally in the mesenchyme according to the guidance molecules they express independent of their OR specificity. After the OSN axons cross into the inner nerve layer of the OB, OR-specific sorting occurs resulting in final convergence into a glomerulus.

Thus, we propose a hierarchical model for the establishment of the glomerular map in which OSN axons sort and regionally segregate prior to the formation of the cribriform plate, at the earliest stages of olfactory nerve formation. This establishes a distinct spatial topography that is maintained as the OSN axons reach the nascent OB (Figure 10). While the OR is critical for axon-induced glomerulogenesis, it appears that OR-specific homotypic fasciculation does not occur until the OSN axons reach the inner nerve layer. This suggests that alternative mechanisms distinct from the OR and those involved with ultimate axonal convergence are responsible for establishing regional topography (Figure 10). Although there may be a constellation of candidate molecular mechanisms, the alterations we report in the regional fasciculation of NRP-1 axons in the AC3 KO mice suggests that NRP-1 may be one such mechanism.

## Abbreviations

AC: adenylyl cyclase; DBA: *Dolichos biflorus *agglutinin; E: embryonic day; GFP: green fluorescent protein; KO: knockout; MM: migratory mass; NQO: NAD(P)H:quinone oxidoreductase; NCAM: neural cell adhesion molecule; NRP: neuropilin; OB: olfactory bulb; OCAM: olfactory cell adhesion molecule; OE: olfactory epithelium; OP: olfactory placode; OR: odor receptor; OSN: olfactory sensory neuron; PBS: phosphate-buffered saline; PONL: presumptive olfactory nerve layer; ROBO2: Roundabout2; WFA: *Wisteria floribunda *agglutinin.

## Competing interests

The authors declare that they have no competing interests.

## Authors' contributions

Conceived and designed the experiments: AMM, LRM and CAG. Performed the experiments: AMM, LRM and D-JZ. Analyzed the data: AMM and CAG. Wrote the paper: AMM LRM, D-JZ, SF and CAG.

## Supplementary Material

Additional file 1 **Figure S1: Single channel localization of NRP1 and NCAM (A', A'', B', B'') from the colocalization shown in (A, B), respectively, and shown in from Figure **2C, D. The arrow in (A) indicates focal localization of NRP1. Click here for file

Additional file 2 **Movie S2: Three-dimensional reconstructions of an E12 sagittal CD-1 embryo rotating from the lateral to the medial view.** This reconstruction depicts regional segregation of NRP-1^+ ^axons in the olfactory nerve. To be viewed in conjunction with Figure 2E. View with Quicktime or Windows Media Player. Click here for file

Additional file 3 **Figure S3: Snapshots of three-dimensional reconstructions of an E12 sagittal CD-1 embryo with surfaces rendered depict regional segregation of NRP-1^+ ^axons in the olfactory nerve.** (A, B) NRP-1/NCAM colocalization (yellow); NCAM (red). (A) Medial view; (B) lateral view. Click here for file

Additional file 4 **Figure S4: Single channel of localization of the NRP1 (A'-D'') from the colocalization shown in (A-D), respectively, and from Figure **5A-B'. Single channel localization of DBA (E'-F'') from colocalization shown in (E, F), respectively, and from Figure 5C, D. (A-B'', E-E'') From ACIII heterozygous control mice. (C-D'', F-F'') From ACIII homozygous KO mice. The nuclear DRAQ5 labeling shown in Figure 5 has been deleted here for clarity. Scale bar = 100 μm in (A, C, E, F) and 25 μm in (B, D). Click here for file

Additional file 5 **Movie S5: P2^+ ^OSN axons do not homotypically fasciculate in the olfactory nerve.** P2-LacZ axons, red; NCAM, blue. To be viewed in conjunction with Figure 7A, A'. View with Quicktime or Windows Media Player. Click here for file

Additional file 6 **Movie S6: M72^+ ^OSN axons are regionally segregated, but do not homotypically fasciculate in the olfactory nerve.** M72-GFP axons, green; NCAM, blue. To be viewed in conjunction with Figure 7B-B'. View with Quicktime or Windows Media Player. Click here for file

Additional file 7 **Movie S7: P2^+ ^OSN axons course along parallel trajectories but do not completely coalesce.** P2-LacZ axons, red; NCAM, blue. To be viewed in conjunction with Figure 9A. View with Quicktime or Windows Media Player. Click here for file

Additional file 8 **Movie S8: High-powered view of M72^+ ^OSN axons that are not homotypically fasciculated in an axon fascicle in the lamina propria.** M72-GFP axons, green; NCAM, blue. To be viewed in conjunction with Figure 9B-B'. View with Quicktime or Windows Media Player. Click here for file

Additional file 9 **Movie S9: M72^+ ^OSN axons are not all contained within the same axon fascicles, and even within axon fascicles are not homotypically fasciculated in the lamina propria.** M72-GFP axons, green; NCAM, blue. To be viewed in conjunction with Figure 9C-C'. View with Quicktime or Windows Media Player. Click here for file

Additional file 10 **Movie S10: High-powered view of P2^+ ^OSN axons that are not homotypically fasciculated in an axon fascicle in the lamina propria.** P2-lacZ axons, red; NCAM, blue. To be viewed in conjunction with Figure 9D-D'. View with Quicktime or Windows Media Player. Click here for file
