# Supplementary figures and images for: Axon fasciculation in the developing olfactory nerve
Source: Neural Dev. 2010 Aug 19;5:20. doi: 10.1186/1749-8104-5-20 (PMC2936880; doi:10.1186/1749-8104-5-20)

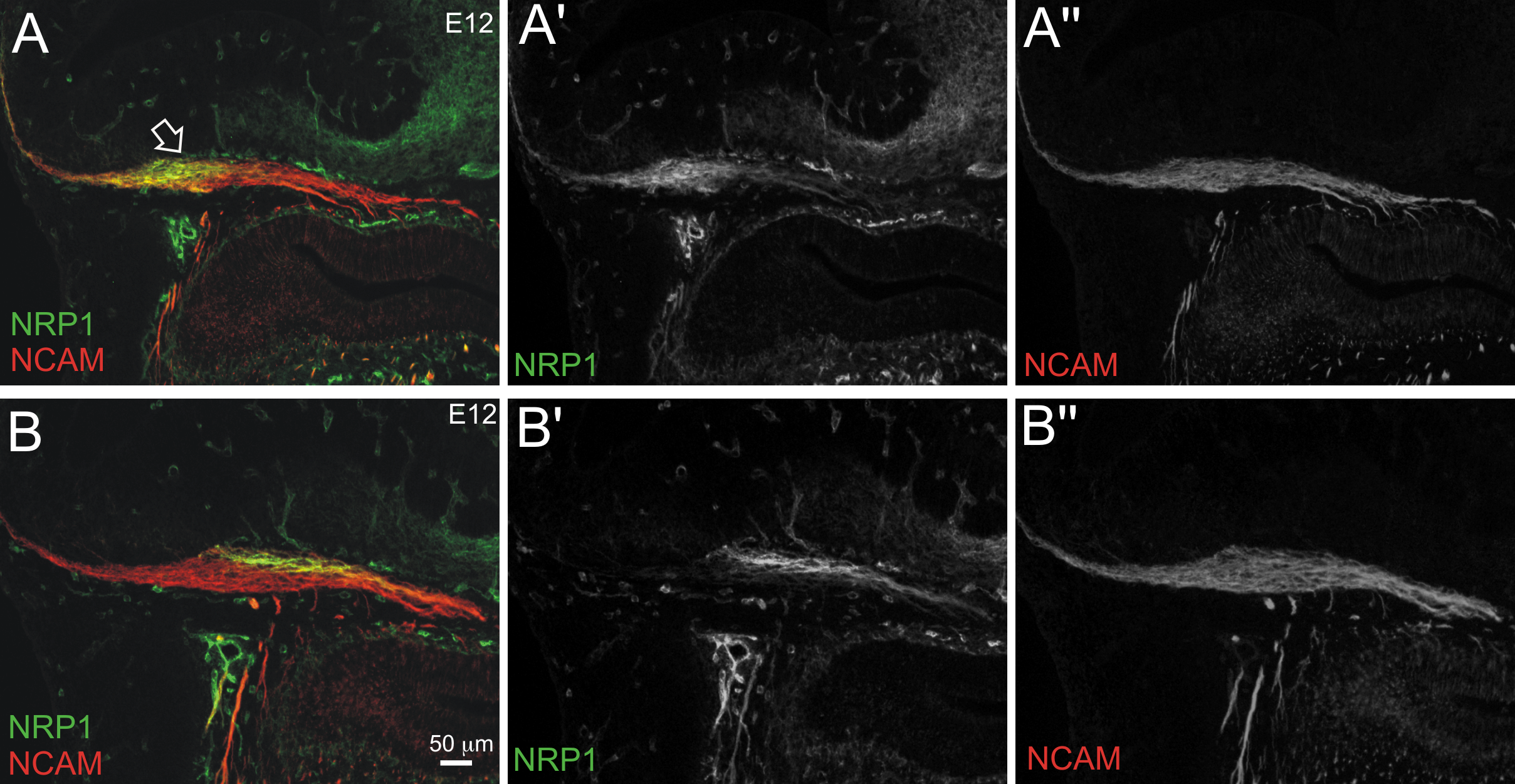

Supplement: Additional file 1 — Figure S1: Single channel localization of NRP1 and NCAM (A', A'', B', B'') from the colocalization shown in (A, B), respectively, and shown in from Figure 2C, D. The arrow in (A) indicates focal localization of NRP1. [file 1749-8104-5-20-S1.TIFF]

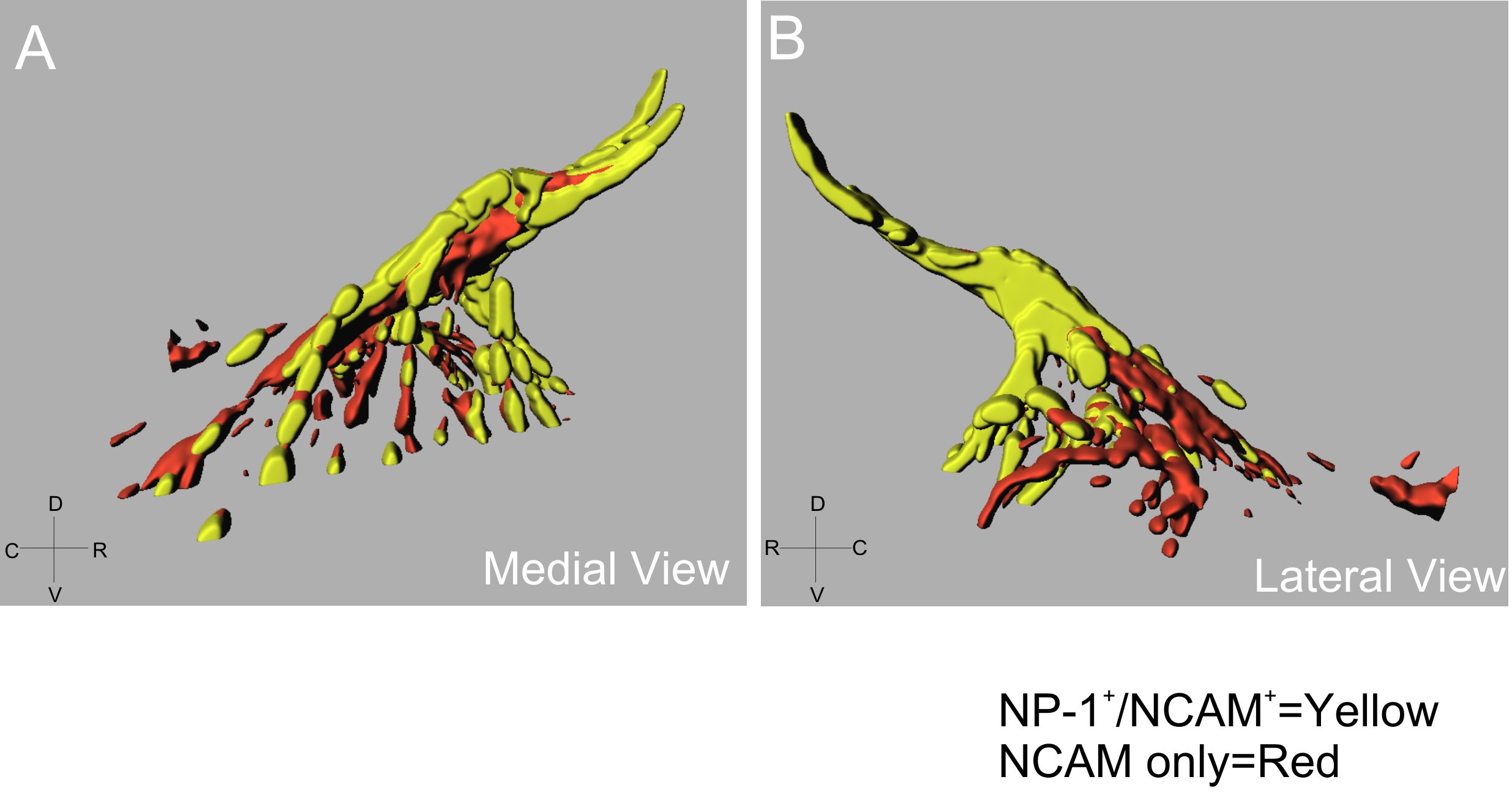

Supplement: Additional file 3 — Figure S3: Snapshots of three-dimensional reconstructions of an E12 sagittal CD-1 embryo with surfaces rendered depict regional segregation of NRP-1+ axons in the olfactory nerve. (A, B) NRP-1/NCAM colocalization (yellow); NCAM (red). (A) Medial view; (B) lateral view. [file 1749-8104-5-20-S3.TIFF]

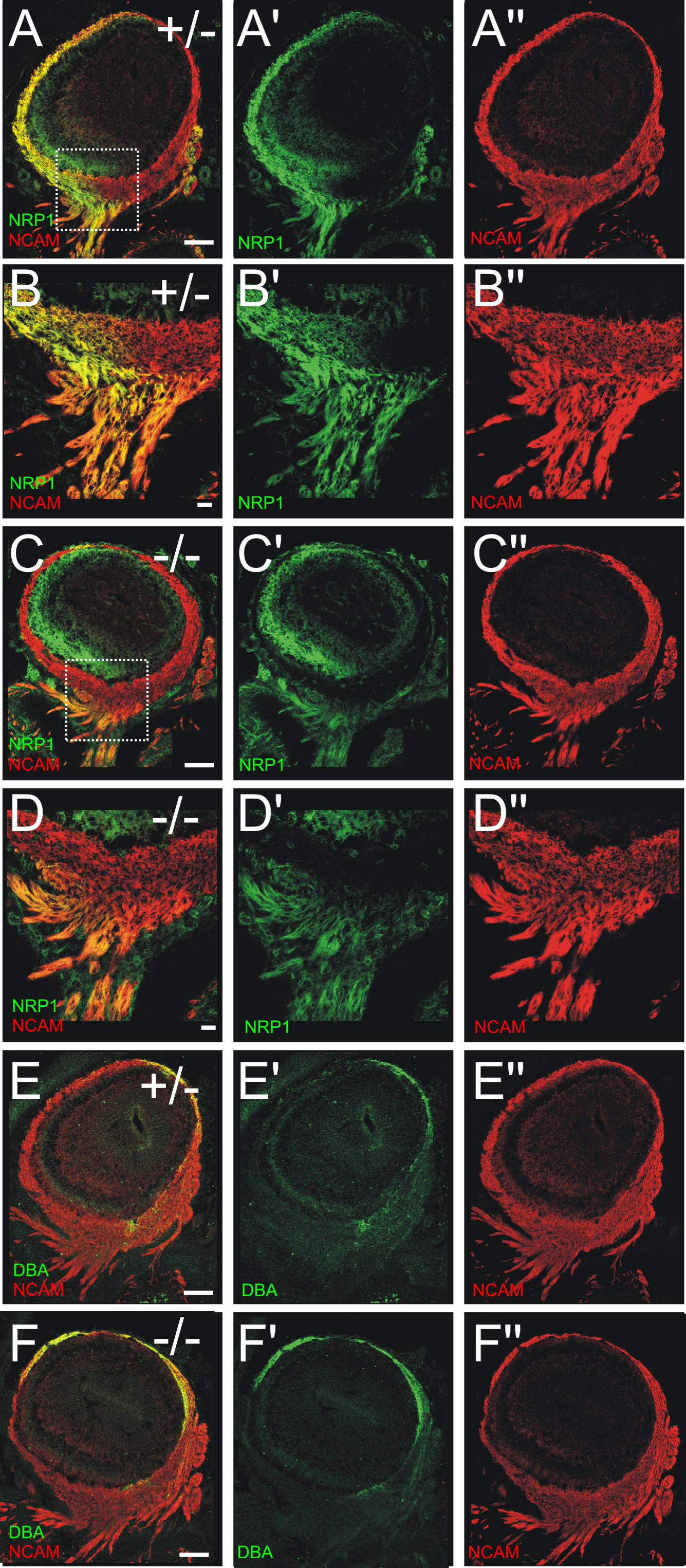

Supplement: Additional file 4 — Figure S4: Single channel of localization of the NRP1 (A'-D'') from the colocalization shown in (A-D), respectively, and from Figure 5A-B'. Single channel localization of DBA (E'-F'') from colocalization shown in (E, F), respectively, and from Figure 5C, D. (A-B'', E-E'') From ACIII heterozygous control mice. (C-D'', F-F'') From ACIII homozygous KO mice. The nuclear DRAQ5 labeling shown in Figure 5 has been deleted here for clarity. Scale bar = 100 μm in (A, C, E, F) and 25 μm in (B, D). [file 1749-8104-5-20-S4.TIFF]
